# Evolving First-Line Endocrine Therapy in HR+/HER2− Metastatic Breast Cancer: CDK4/6 Inhibition, Biomarker-Guided Strategies and Emerging Therapeutic Paradigms

**DOI:** 10.3390/curroncol33070421

**Published:** 2026-07-14

**Authors:** Hikmat Abdel-Razeq, Baha Sharaf

**Affiliations:** 1 Section of Hematology and Medical Oncology, Department of Internal Medicine, King Hussein Cancer Center, Amman 11941, Jordan; 2 School of Medicine, The University of Jordan, Amman 11942, Jordan

**Keywords:** endocrine therapy, HR-positive, metastatic breast cancer, CDK4/6 inhibitors, ESR1, ctDNA

## Abstract

Hormone receptor-positive (HR+), human epidermal growth factor receptor 2-negative (HER2–) metastatic breast cancer (MBC) is the most prevalent subtype of advanced breast cancer and is predominantly driven by estrogen receptor (ER) signaling. Endocrine therapy (ET) has become the backbone of first-line treatment; however, both intrinsic and acquired resistance limit long-term disease control. The introduction of cyclin-dependent kinase 4/6 (CDK4/6) inhibitors has fundamentally reshaped the therapeutic landscape even in subsets of patients with aggressive or symptomatic visceral metastatic disease. Advances in molecular profiling have also enabled more precise, adaptive therapy. Circulating tumor DNA (ctDNA)-based liquid biopsy now allows real-time detection of emerging resistance mutations, particularly in ESR1. Additionally, patients with *PIK3CA*-mutated tumors who had progressed on or within 12 months of completing adjuvant ET and had no prior systemic therapy for metastatic disease had better treatment outcomes when treated with the PI3K inhibitor inavolisib in combination with palbociclib and fulvestrant. Together, these developments mark a shift from fixed treatment sequencing toward a more dynamic, biomarker-driven approach in first-line HR+/HER2– MBC. Integration of CDK4/6 inhibitors with next-generation endocrine agents and liquid biopsy-guided therapy offers the potential to delay resistance, improve survival outcomes, and individualize treatment.

## Simple Summary

Hormone receptor-positive, HER2-negative metastatic breast cancer, the most common type of advanced breast cancer, depends on estrogen to grow. Treatments that block this hormone remain the basis of care, especially when combined with newer drugs (CDK4/6 inhibitors) that slow cancer cell division and significantly prolong survival. However, most cancers eventually become resistant to this therapy. Recent advances are helping physicians stay ahead of this resistance. Blood-based tests (liquid biopsies) can now detect early genetic changes in the tumor, allowing treatment to be adjusted before the disease worsens. In addition, targeted therapies can be added for patients with specific mutations (such as *PIK3CA*), further improving outcomes. Altogether, care is moving toward a more personalized and adaptive approach, aiming to extend disease control, delay the need for chemotherapy, and maintain quality of life.

## 1. Introduction

Metastatic breast cancer (MBC) remains a major cause of morbidity and mortality worldwide [1]. Hormone receptor-positive (HR+) disease, defined by estrogen receptor (ER) and/or progesterone receptor (PR) positivity and absence of human epidermal growth factor receptor 2 (HER2) overexpression, represents the predominant subtype and is highly dependent on estrogen signaling for tumor growth [2,3]. Endocrine therapy (ET) has been central to managing these tumors, exploiting blockade of estrogen production or receptor signaling to control disease progression [4]. Historically, single-agent aromatase inhibitors (AI), tamoxifen, or fulvestrant provided modest improvement in survival [5]. However, the therapeutic landscape transformed with the advent of cyclin-dependent kinase 4/6 (CDK4/6) inhibitors in combination with ET, which significantly improved progression-free survival (PFS), overall survival (OS), and quality of life (QOL) in multiple randomized trials [6,7]. Despite these advances, resistance remains universal, and novel agents targeting ER signaling and key resistant pathways are under intense development. Recent progress in genomic profiling, circulating tumor DNA (ctDNA) monitoring, and the introduction of next-generation estrogen receptor degraders (SERDs) and pathway-directed combinations have initiated a shift from uniform treatment algorithms toward biomarker-driven precision therapy.

This review critically appraises the latest advances in first-line endocrine therapy for HR+/HER2− MBC, focusing on biomarker-guided therapies, mechanism-driven combinations, new drug approvals and emerging concepts in treatment sequencing.

## 2. Methods

In preparing this review, our aim was to create a coherent and clinically meaningful narrative that reflects the rapid evolution of first-line endocrine therapy in HR-positive, HER2-negative metastatic breast cancer. We performed a comprehensive literature search in PubMed/MEDLINE, EMBASE, and ClinicalTrials.gov for studies published between 2010 and January 2026, using keywords related to endocrine resistance, CDK4/6 inhibition, ctDNA, *ESR1* mutations, *PIK3CA*, and SERDs. We also reviewed abstracts from major oncology meetings including the American Society of Clinical Oncology (ASCO), the European Society of Medical Oncology (ESMO) and the San Antonio Breast Cancer Symposium (SABCS), to ensure inclusion of emerging data that may not yet be published but already influence clinical practice. Priority was given to randomized trials, biomarker-driven studies, and high-quality translational research. This review does not follow a formal systematic methodology; instead, it is designed to integrate the most relevant and timely evidence to assist clinicians in navigating increasingly complex treatment choices in HR+/HER2– metastatic disease.

## 3. Mechanisms of Endocrine Resistance and Therapeutic Implications

Despite major improvements in outcomes with endocrine therapy (ET)-based combinations, resistance, either intrinsic or acquired, remains inevitable in most patients with HR+/HER2– MBC. Multiple, often overlapping molecular mechanisms drive this phenomenon, including ER alterations, activation of alternative signaling pathways, and tumor clonal evolution under therapeutic pressure [8].

### 3.1. ESR1 Mutations

The *ESR1* gene encodes estrogen receptor-alpha (ERα), a central driver of HR-positive breast cancer biology. Somatic mutations within the receptor’s ligand-binding domain, most commonly at residues Y537 and D538, represent a key mechanism of acquired resistance in HR-positive MBC [9]. These mutations induce conformational changes that stabilize the receptor in an active state that leads to ligand-independent transcriptional signaling and tumor growth despite estrogen deprivation [10]. *ESR1* mutations are rarely encountered in treatment-naïve primary tumors; however, they emerge in up to 40% of patients following treatment with aromatase inhibitors (AIs) [11].

These resistant clones can be heterogeneous within the tumor and often subclonal; as such, single-site tissue biopsies frequently underestimate their presence [12,13]. Liquid biopsy analysis of circulating tumor DNA (ctDNA), performed using highly sensitive digital droplet PCR (ddPCR) or next-generation sequencing (NGS), usually provides a more comprehensive and dynamic assessment of evolving tumor genomics across several metastatic sites [14]. Recently published clinical trials have linked ESR1 mutation to many therapeutic interventions. Current clinical guidelines increasingly recommend ESR1 testing at the time of disease progression on ET; while emerging prospective trials such as PADA-1 and SERENA-6 have introduced a preemptive strategy of molecular monitoring in which detection of an expanding ESR1 clone in ctDNA can prompt an early therapeutic switch [15].

### 3.2. Activation of Alternative Signaling Pathways

Resistance to ET is frequently mediated by activation of estrogen-independent growth and survival pathways, most notably the PI3K/AKT/mTOR pathway and dysregulation of the cyclin D–CDK4/6–Rb axis [16]. Activating *PIK3CA* mutations, present in approximately 40% of HR+ breast cancers, promote downstream AKT and mTOR signaling, enabling tumor proliferation despite ER blockade [17]. Understanding these pathways has led to the development of alpelisib (PI3K inhibitor) and everolimus (mTOR inhibitor) [18,19]. In parallel, alterations in cell-cycle control, including CDK4/6 activation, cyclin D1 amplification and RB pathway disruption, drive further resistance. This biological rationale supports the observed clinical benefit with CDK4/6 inhibitors when combined with ET, restoring endocrine sensitivity and delaying resistance [20].

Importantly, PI3K/AKT pathway activation also directly influences cell-cycle progression by promoting CDK4/6-mediated G1–S transition and by phosphorylating and inactivating the RB protein, thereby bypassing the cell-cycle checkpoint that CDK4/6 inhibitors are designed to restore. This mechanistic crosstalk between PI3K/AKT signaling and cell-cycle regulation provides additional biological rationale for combining PI3K pathway inhibitors with CDK4/6 inhibitors and endocrine therapy [21].

### 3.3. Tumor Heterogeneity and Clonal Evolution

Breast cancer is not a static entity but rather a dynamic ecosystem shaped by selective pressure from therapy. Intra-tumoral and inter-site heterogeneity means that resistant subclones may co-exist with sensitive populations from the outset, or emerge under treatment pressure through clonal selection [22,23].

Longitudinal monitoring of tumor clonal evolution using ctDNA enables dynamic tracking of these changes over time, supporting an emerging paradigm of biomarker-guided, adaptive therapeutic intervention. Identifying expanding resistant clones at a molecular level offers the potential to refine treatment sequencing months before clinical or radiological progression is encountered [23,24,25].

### 3.4. Emerging Resistance Biomarkers

Beyond ESR1 and *PIK3CA*, a broader landscape of molecular alterations has been implicated in endocrine and CDK4/6 inhibitor resistance, although most are not yet routinely actionable in the first-line setting. Loss of RB1, which encodes the retinoblastoma protein, directly bypasses the G1/S checkpoint and confers resistance to CDK4/6 inhibitors [26]. Alterations in FGFR1/2 amplification or mutation activate alternative mitogenic signaling independent of ER. PTEN loss and AKT1 activating mutations hyperactivate the PI3K/AKT axis through *PIK3CA*-independent mechanisms [27]. TP53 mutations are associated with aggressive phenotypes, reduced endocrine sensitivity, and poorer prognosis. Comprehensive genomic profiling at baseline and at progression increasingly identifies these alterations, and while prospective data supporting specific therapeutic interventions based on these biomarkers in the first-line setting remain limited, their characterization may inform future trial eligibility and sequencing decisions [28].

## 4. Initial Assessment and Biomarker Testing

A comprehensive biologic assessment at the time of diagnosis of metastatic HR-positive, HER2-negative breast cancer is essential to guide optimal treatment selection and sequencing. Whenever clinically feasible, biopsy of a metastatic lesion should be performed to reconfirm ER, PR, and HER2 status, as discordance in receptor status between the primary tumor and metastatic sites in approximately 10–30% of patients [29,30].

Beyond standard immunohistochemistry, genomic profiling has become a critical component of baseline assessment, particularly for the detection of actionable alterations such as ESR1, *PIK3CA*, AKT1, and PTEN. These biomarkers not only provide prognostic information but also directly inform therapeutic decision-making [31,32]. For example, ESR1 mutations predict resistance to aromatase inhibitors and favor the use of SERDs or ER degraders, while detection of *PIK3CA* mutations identifies patients who may benefit from PI3Kα inhibitors such as inavolisib or alpelisib, as will be addressed in this manuscript [33,34].

## 5. Current Standard of Care in First-Line HR+/HER2 MBC

### 5.1. CDK4/6 Inhibitors Plus Endocrine Therapy: A Paradigm Shift

The integration of CDK4/6 inhibitors with ET has transformed first-line therapy for HR+/HER2 MBC [35]. Palbociclib [36], ribociclib [37], and abemaciclib [38] inhibitors of CDK4 and CDK6, key cell cycle regulatory kinases, arrest tumor cells in the G1 phase, amplifying the antiproliferative effects of ET [39]. Multiple phase 3 randomized trials demonstrated that adding a CDK4/6 inhibitor to AI or fulvestrant significantly extends PFS compared to ET alone and, importantly, yields OS benefits without compromising quality of life [40,41]. Multiple meta-analyses confirm the superiority of the combined approach over ET monotherapy, establishing CDK4/6 inhibitors plus ET as the standard first-line regimen in postmenopausal patients and many premenopausal patients who also receive ovarian suppression [42,43,44]. Current international guidelines (NCCN, ASCO) continue to recommend CDK4/6 inhibitors with an aromatase inhibitor or fulvestrant as first-line therapy for patients not requiring immediate chemotherapy [45,46,47].

#### 5.1.1. Clinical Evidence Supporting CDK4/6 Inhibitors in First-Line Therapy

The clinical value of CDK4/6 inhibitors in the first-line setting has been established through multiple large, randomized phase 3 trials. Three agents, palbociclib, ribociclib, and abemaciclib, have each been evaluated in combination with AI or fulvestrant as initial therapy for HR+/HER2− MBC.

Across trials such as PALOMA-2 [48], MONARCH-3 [49,50], and MONALEESA-2 [51,52], the addition of a CDK4/6 inhibitor to an aromatase inhibitor nearly doubled progression-free survival (PFS) compared with endocrine therapy alone. Median PFS improved from approximately 12–14 months with AI monotherapy to 24–28 months with combination therapy. These results were consistent across patient subgroups, including those with visceral metastases, de novo metastatic disease, or prior adjuvant endocrine therapy.

Beyond disease control, CDK4/6 inhibitors have redefined survival expectations. Ribociclib has demonstrated consistent and statistically significant overall survival benefit in first-line and later-line settings across the MONALEESA trial program [6,53]. Abemaciclib has also shown OS benefit in MONARCH-2 [54,55], while palbociclib, despite robust PFS gains, has not shown a reproducible OS advantage in randomized trials [56]. These data have influenced guideline recommendations, with ribociclib often considered the preferred CDK4/6 inhibitor. Table 1 details differences in the treatment outcomes of the three CDK4/6 inhibitors.

The absence of a statistically significant OS benefit with palbociclib stands in sharp contrast to both ribociclib and abemaciclib, and several converging explanations have been proposed. In PALOMA-2, after a median follow-up of 90.1 months, the longest of any first-line CDK4/6 inhibitor trial, median OS was 53.9 versus 51.2 months (HR 0.956; *p* = 0.34), a null result that cannot be attributed to insufficient follow-up time. At the pharmacological level, palbociclib has similar binding affinity for CDK4 and CDK6, whereas ribociclib and abemaciclib have approximately fivefold and ninefold greater affinity for CDK4/cyclin D3, respectively, and since CDK4 is the dominant driver of Rb phosphorylation and G1/S transition in ER-positive breast cancer, this CDK4-selective advantage may translate to deeper and more durable tumor cell cycle suppression with ribociclib and abemaciclib. Compounding the pharmacological difference, 26.7% of patients in the PALOMA-2 placebo arm received a subsequent CDK4/6 inhibitor; 89.7% of whom received palbociclib, compared with only 11.8% in the combination arm, an asymmetric rescue of the control arm that would be expected to substantially compress the OS separation between treatment groups. Additionally, abemaciclib’s continuous twice-daily dosing schedule, in contrast to the 3-weeks-on/1-week-off intermittent schedule shared by palbociclib and ribociclib, may sustain more durable CDK4 suppression and more pronounced induction of cellular senescence throughout the treatment cycle. In MONALEESA-2, by comparison, the OS benefit of 63.9 versus 51.4 months (HR 0.76; *p* = 0.008) was achieved at a median follow-up of 80 months, a shorter observation period than PALOMA-2, making pharmacological and trial-design differences, rather than follow-up maturity, the most plausible explanations for the divergent OS results. Critically, however, no head-to-head randomized trial has compared the three agents, and the absence of an OS signal with palbociclib does not constitute proof of inferiority; palbociclib retains an important clinical role, most notably as the backbone of the FDA-approved inavolisib triplet in *PIK3CA*-mutated disease [45,46,47,48,49,50,51,52].

#### 5.1.2. Choice of Endocrine Partner: Evidence from the PARSIFAL Trial

In the Phase 2 PARSIFAL trial [59], the optimal endocrine backbone for CDK4/6 inhibition in the first-line treatment of HR+, HER2-negative advanced breast cancer was evaluated by randomizing 486 postmenopausal women to receive palbociclib in combination with either letrozole or fulvestrant. After a median follow-up of 32 months, results showed no statistically significant difference in PFS between the two treatment arms, with median PFS recorded at 27.9 months for the palbociclib–letrozole group versus 32.8 months for the palbociclib–fulvestrant group, yielding a hazard ratio (HR) of 1.13 (95% CI, 0.85–1.50). Additionally, there were no meaningful differences in objective response rate, overall survival, or safety profiles between the two combinations. The PARSIFAL remains the largest randomized trial directly comparing an AI to a SERD as the endocrine backbone for CDK4/6 inhibitor therapy, supporting the continued use of AIs as an effective and pragmatic option while reserving fulvestrant for specific clinical scenarios such as early relapse on adjuvant therapy or cases of ESR1-mediated resistance.

#### 5.1.3. CDK4/6 Inhibitors in Visceral Metastasis

Clinical definition of visceral crisis: Before reviewing the trial evidence, it is important to distinguish ‘visceral metastases’ from ‘visceral crisis,’ as confusion between these terms can lead to misapplication of trial results. Visceral crisis (or visceral organ dysfunction) is defined, per ESMO consensus guidelines, as severe organ dysfunction due to metastatic disease—assessed by symptoms, signs, and laboratory and/or imaging findings—that requires rapid and effective therapy to avoid rapid deterioration and life-threatening organ failure. Clinically, this typically includes rapidly deteriorating performance status, lymphangitic pulmonary carcinomatosis causing respiratory compromise, severe hepatic dysfunction (bilirubin >3× ULN or hepatic encephalopathy), or situations requiring urgent tumor burden reduction to prevent imminent organ failure. Critically, the mere presence of liver, lung, or other visceral organ metastases does not constitute a visceral crisis. This distinction is essential for appropriately applying the trial results discussed below.

##### The RIGHT Choice Trial

The RIGHT Choice trial was the first prospective randomized study comparing first-line ribociclib + endocrine therapy versus physician-choice combination chemotherapy specifically in patients with aggressive HR+/HER2 MBC, including those with symptomatic visceral metastases and visceral crisis, a group often managed with upfront chemotherapy [60]. However, patients with life-threatening organ dysfunction, i.e., patients with severe hepatic dysfunction, bilirubin >1.5× upper limit of normal values, were excluded.

In this study, 222 pre/perimenopausal women with clinically aggressive metastatic disease, including those with symptomatic visceral metastases and rapidly progressing disease, were randomized to ribociclib plus letrozole/anastrozole plus goserelin vs. investigator-chosen combination chemotherapy. At a median follow-up time of 37.0 months, the median PFS was 21.8 months with ribociclib + ET vs. 12.8 months with chemotherapy (HR 0.61; *p* = 0.003). The overall response rates and the median time to response in the ribociclib arm were 66.1% and 4.9 months compared to 61.8% and 3.2 months in the chemotherapy arm (hazard ratio, 0.76 [95% CI, 0.55 to 1.06]). Ribociclib + ET had a more favorable tolerability profile with fewer symptomatic adverse events and fewer treatment discontinuations. As such, the RIGHT Choice challenged the long-held belief that aggressive or visceral disease necessarily requires upfront chemotherapy by showing that ribociclib plus ET could provide superior PFS with less toxicity, supporting its ‘chemotherapy-sparing’ role even in high-burden visceral disease.

##### The PADMA Trial

Although not yet fully published in peer-reviewed form, emerging data from the PADMA study reinforces the value of CDK4/6 inhibitor-based therapy versus mono chemotherapy in high-risk metastatic disease. Patients with indications for chemotherapy (e.g., visceral metastasis, rapid progression) randomized to palbociclib plus ET showed significantly better outcomes in time to treatment failure (TTF) and PFS compared with mono chemotherapy (HR 0.45 for PFS in favor of CDK4/6 plus ET) [61].

While these results involve palbociclib rather than ribociclib, they support a broader paradigm in which CDK4/6 inhibitors plus ET may outperform chemotherapy even in traditionally high-risk visceral disease settings [62].

##### The ABIGAIL Trial

Complementing the findings of the RIGHT Choice study, the ABIGAIL trial investigated whether upfront abemaciclib plus ET could serve as a more effective alternative to induction chemotherapy in patients with HR+/HER2− advanced breast cancer presenting with aggressive disease. This is a phase 2 multicenter study that randomized 162 patients, including those with symptomatic visceral metastases and high-grade tumors, to receive either abemaciclib plus ET (letrozole or fulvestrant) or weekly paclitaxel for 12 weeks [63].

Based on primary analysis presented at the European Society of Medical Oncology (ESMO) congress 2024, the trial met its primary endpoint with a superior 12-week objective response rate (ORR) of 58.8% for the abemaciclib arm compared to 40.2% in the induction chemotherapy arm, *p* = 0.019. This significant improvement was achieved with a favorable safety profile, with less alopecia (5% vs. 39%), peripheral neuropathy (0% vs. 28%), and drug discontinuation (6.3% vs. 27%), but with a higher incidence of diarrhea (68% vs. 23%) [64]. Table 2 summarizes the three discussed studies.

#### 5.1.4. Tolerability and Quality of Life

A major strength of CDK4/6 inhibitor-based therapy is its favorable balance between efficacy and tolerability. While neutropenia is common with palbociclib and ribociclib and diarrhea with abemaciclib, these toxicities are generally manageable with dose adjustments and supportive care. Importantly, patients receiving CDK4/6 inhibitors maintain better quality of life, experience fewer hospitalizations, and have longer chemotherapy-free intervals compared with those receiving cytotoxic therapy [65,66,67].

### 5.2. Fulvestrant

Prior to the introduction of CDK4/6 inhibitors, or on rare occasions where patients are not suitable for these drugs, fulvestrant, a selective estrogen receptor degrader, was a reasonable treatment option. Fulvestrant has been formally evaluated as a first-line endocrine therapy for patients with HR-positive, HER2-negative MBC in two pivotal randomized trials. The phase 3 FALCON trial enrolled 462 postmenopausal women with HR+/HER2− locally advanced or metastatic breast cancer who had received no prior endocrine therapy for advanced disease and were randomized to receive fulvestrant 500 mg or anastrozole 1 mg. Patients with both visceral and non-visceral disease were eligible, although none had prior exposure to AI in the metastatic setting. Fulvestrant significantly improved PFS compared with anastrozole (median 16.6 vs. 13.8 months; HR 0.80; *p* = 0.049). Notably, the magnitude of benefit was greatest in patients with non-visceral disease, in whom median PFS was 22.3 months with fulvestrant versus 13.8 months with anastrozole. The safety profile was comparable between arms, supporting its regulatory approval as a first-line endocrine monotherapy in selected patients [68,69].

These results were built upon the earlier phase 2 FIRST trial, which enrolled 205 postmenopausal women with HR^+^ advanced breast cancer who were endocrine therapy-naïve in the metastatic setting and randomized them to fulvestrant 500 mg or anastrozole. Although originally designed to assess clinical benefit rate as its primary endpoint, FIRST provided a striking efficacy signal: fulvestrant significantly prolonged time to progression and was associated with a numerical improvement in overall survival, with median OS of 54.1 months versus 48.4 months for anastrozole (HR 0.70) [70,71]. Together, FIRST and FALCON established fulvestrant 500 mg as a biologically and clinically superior ER–targeting strategy compared with aromatase inhibition in endocrine-sensitive HR+ metastatic breast cancer, providing the foundation for its subsequent integration as a backbone for combination regimens with CDK4/6 inhibitors and next-generation SERDs.

Meta-analyses of first-line endocrine monotherapies have further suggested that fulvestrant 500 mg ranks among the most effective single-agent options for HR-positive advanced disease in terms of objective response rates and time to progression, alongside AI [72]. While contemporary practice increasingly integrates CDK4/6 inhibition with fulvestrant or AI in the first-line setting, these randomized data establish fulvestrant monotherapy as a validated first-line endocrine strategy in appropriately selected patients [73].

Fulvestrant was also tried in combination with AI. In the SWOG S0226, postmenopausal women with HR+ metastatic breast cancer were randomized to receive anastrozole alone or anastrozole plus fulvestrant as first-line therapy. The combination significantly improved PFS and long-term follow-up also showed a modest OS benefit (median 49.8 vs. 42.0 months; HR 0.82). The benefit was most pronounced in women without prior adjuvant endocrine therapy [74].

### 5.3. New and Emerging First-Line Therapeutic Strategies

#### 5.3.1. Next-Generation SERDs

SERDs have emerged as a transformative strategy in the endocrine treatment of HR+, HER2-negative MBC by directly targeting ER for degradation rather than merely antagonizing its activity [75]. First-generation SERDs such as fulvestrant demonstrated proof of principle by degrading ER and achieving clinical benefit but were limited by intramuscular administration and suboptimal pharmacokinetics. Second-generation orally bioavailable SERDs were thus developed to improve receptor degradation, convenience of dosing, and antitumor efficacy, particularly in the setting of ESR1 mutations that drive ligand-independent ER signaling and resistance to aromatase inhibitors and other endocrine therapies [76]. Meta-analyses and prospective studies confirm that oral SERDs can extend PFS compared with physician’s choice therapies in HR+/HER2− metastatic breast cancer, with the benefit most pronounced in patients with ESR1-mutant disease [77].

Giredestrant is an investigational oral SERD that has demonstrated early clinical activity in ER-positive, HER2-negative advanced breast cancer, including in phase Ia/b dose-escalation studies where it showed tolerability and preliminary antitumor effects alone and with palbociclib in locally advanced/metastatic settings [78].To formally evaluate its potential as first-line ET in MBC, the phase 3 persevERA breast cancer trial has been conducted, randomizing patients with ER+/HER2-negative locally advanced or metastatic disease to giredestrant plus palbociclib versus letrozole plus palbociclib as initial treatment. Although the study demonstrated a numerical improvement in PFS compared with letrozole plus palbociclib, it did not achieve its primary endpoint of a statistically significant PFS benefit, highlighting the ongoing challenges of improving outcomes beyond current CDK4/6 inhibitor–based endocrine therapy standards [79]. Although giredestrant has shown promising outcomes in other settings (e.g., improving PFS with everolimus in advanced disease [80], and demonstrating superiority over standard endocrine therapy in early breast cancer in the lidERA adjuvant trial), it is not yet established or approved as standard first-line endocrine therapy for MBC at present [81].

Camizestrant, another oral SERD, has demonstrated promising antitumor activity in ER-positive, HER2-negative MBC, including in patients with prior CDK4/6 inhibitor therapy and ESR1 mutations (SERENA-1 and SERENA-2 trials) [82,83]. Building on this, the phase-3 SERENA-6 trial adopted a novel ctDNA-guided design in patients receiving first-line AI plus CDK4/6 inhibitor therapy: upon detection of emerging ESR1 mutations ahead of radiographic progression, patients were randomized to switch to camizestrant with continued CDK4/6 inhibition versus continuation of the aromatase inhibitor; details will be discussed in later sections [84].

Imlunestrant is a third oral SERD that has advanced through the EMBER clinical trial program. Imlunestrant selectively degrades ERα and has shown potent activity in preclinical models with ESR1 mutations. In the phase 2 EMBER trial, imlunestrant monotherapy demonstrated clinical activity in patients with HR+/HER2− advanced breast cancer who had received prior endocrine therapy, with a favorable tolerability profile. The pivotal phase 3 EMBER-3 trial compared imlunestrant monotherapy versus imlunestrant plus abemaciclib versus investigator’s choice endocrine therapy in patients with prior endocrine therapy exposure; results presented at ASCO 2024 demonstrated a significant improvement in PFS with imlunestrant over standard endocrine therapy, with the benefit most pronounced in patients with ESR1-mutant disease. These data position imlunestrant as another active oral SERD with a distinct combination profile, and ongoing studies including EMBER-4 in the adjuvant setting will further define its clinical role. Collectively, giredestrant, camizestrant, and imlunestrant represent a new generation of oral SERDs likely to reshape endocrine therapy sequencing in the coming years [72,85,86].

#### 5.3.2. Targeted Combinations Addressing Resistant Pathways

Despite major advances with CDK4/6 inhibitors combined with endocrine therapy, the majority of tumors ultimately develop adaptive resistance through activation of parallel survival pathways, among which the PI3K–AKT–mTOR axis is the most frequently dysregulated. Activating *PIK3CA* mutations, present in approximately 35–40% of HR+ breast cancers, drive estrogen-independent growth, promote resistance to aromatase inhibitors and SERDs, and are strongly associated with reduced endocrine sensitivity and earlier disease progression [87]. The clinical relevance of targeting this pathway was first firmly established by the pivotal phase 3 SOLAR-1 trial, which demonstrated that the addition of the PI3Kα inhibitor alpelisib to fulvestrant significantly prolonged PFS in patients with *PIK3CA*-mutated disease, while conferring no benefit in the wild-type population, thereby validating *PIK3CA* as a predictive biomarker and cementing molecular testing as a prerequisite for treatment selection. However, the conduct of SOLAR-1 prior to the widespread incorporation of CDK4/6 inhibitors and the metabolic toxicities associated with earlier-generation PI3K inhibitors restricted their clinical adoption primarily to later-line settings [88,89,90]. This created a strong rationale for the development of next-generation, mutant-selective PI3Kα inhibitors capable of delivering deeper and more durable pathway suppression with improved safety, thereby enabling integration into frontline combination strategies alongside CDK4/6 inhibitors and endocrine therapy. In this context, inavolisib represents a major evolution in PI3K-targeted therapy, specifically designed to exploit oncogenic *PIK3CA* dependence while minimizing systemic toxicity, setting the stage for the landmark results observed in the INAVO120 trial [91].

##### PI3K Alpha Inhibitors and Triple Combinations: Inavolisib

In the Phase 3 INAVO120 trial, 325 patients with *PIK3CA*-mutated, hormone receptor–positive, HER2-negative advanced breast cancer who had progressed on or within 12 months of completing adjuvant endocrine therapy and had no prior systemic therapy for metastatic disease were randomized to receive inavolisib in combination with palbociclib and fulvestrant (n = 161) or placebo plus palbociclib and fulvestrant (n = 164) [83]. After a median follow-up of 34.2 months, the addition of inavolisib significantly improved clinical outcomes: median progression-free survival was prolonged to 17.2 months versus 7.3 months with placebo (hazard ratio [HR] 0.42; 95% CI 0.32–0.55), and median overall survival was 34.0 months compared with 27.0 months in the control arm (HR 0.67; 95% CI 0.48–0.94), crossing the prespecified boundary for statistical significance [92]. Objective response rates were markedly higher with inavolisib (62.7% vs. 28.0%, *p* < 0.0001), and the addition of inavolisib also substantially delayed time to chemotherapy (35.6 months vs. 12.6 months; HR 0.43), underscoring durable benefit across key endpoints. The safety profile was consistent with known toxicities of PI3K inhibition and the backbone regimen, with higher rates of hyperglycemia and stomatitis but no new safety signals and low discontinuation rates due to adverse events (6.8% vs. 0.6%). In 2024, the U.S. Food and Drug Administration approved inavolisib in combination with palbociclib and fulvestrant to treat people with HR-positive, HER2-negative advanced breast cancer with a *PIK3CA* mutation that has grown following treatment with hormone therapy as discussed above [93].

Despite its transformative Overall Survival (OS) benefit, the INAVO120 trial’s generalizability is constrained by its stringent inclusion criteria, particularly the exclusion of patients with pre-existing diabetes or even mild glucose intolerance (HbA1c ≥ 6.0%). Furthermore, the study’s focus on a palbociclib backbone leaves a data void regarding combinations with other CDK4/6 inhibitors, such as ribociclib or abemaciclib, which are increasingly favored in frontline settings, so clinicians must decide whether to “mix and match” (off-label) or stick to the trial’s exact triplet, which uses the numerically “weaker” CDK4/6 inhibitor (palbociclib). Given that the cohort was almost entirely naive to prior CDK4/6 inhibition, having relapsed early during or within 12 months of adjuvant endocrine therapy, the clinical utility of this triplet in an increasingly “pre-treated” population remains an area of active investigation through trials like INAVO121. Additionally, the regimen significantly delayed the time to subsequent chemotherapy by approximately two years (35.6 vs. 12.6 months), reinforcing its efficacy while highlighting a substantial treatment burden; nearly 91% of patients experienced grade 3/4 adverse events, and discontinuation rates due to toxicity were notably higher in the triplet arm (6.8% vs. 0.6%). Detailed outcomes are summarized in Table 3 [92].

For *PIK3CA*-mutated patients who are ineligible for or unable to tolerate the inavolisib triplet, most commonly due to pre-existing diabetes or glucose intolerance, given the INAVO120 exclusion criterion of HbA1c ≥ 6.0%, the recommended first-line alternative is de-escalation to a standard CDK4/6 inhibitor plus endocrine therapy doublet, with ribociclib preferred on the basis of its demonstrated OS benefit. It is important to emphasize that neither alpelisib plus fulvestrant (SOLAR-1) [89] nor capivasertib plus fulvestrant (CAPItello-291) [94] represents a first-line alternative in this setting: both agents are validated exclusively in patients who have progressed on prior endocrine-based therapy and were not studied in the frontline context, placing them firmly in the sequential second-line space following CDK4/6 inhibitor progression. The evolving post-CDK4/6 inhibitor landscape in *PIK3CA*-mutated disease is being directly addressed by the ongoing Phase III INAVO121 trial (NCT05646862), which compares inavolisib plus fulvestrant versus alpelisib plus fulvestrant in patients with *PIK3CA*-mutated HR+/HER2− advanced breast cancer who have progressed on prior CDK4/6 inhibitor plus endocrine therapy. INAVO121 is therefore not designed to address first-line triplet intolerance; rather, it poses the clinically important question of whether the next-generation, mutant-selective PI3Kα inhibitor inavolisib can deliver superior efficacy and tolerability over alpelisib in the second-line PI3K-directed setting, a comparison that could establish inavolisib as the preferred PI3K-targeting agent across both lines of therapy in *PIK3CA*-mutated disease.

In summary, the addition of inavolisib to palbociclib and fulvestrant more than doubled PFS, produced a statistically significant 7-month improvement in overall survival, more than doubled objective responses, and delayed the need for chemotherapy by nearly two years, establishing this regimen as a highly effective first-line strategy for *PIK3CA*-mutated HR+/HER2− metastatic breast cancer.

#### 5.3.3. Unresolved Controversies and Implementation Challenges

Despite the compelling efficacy data, several critical clinical questions remain unresolved as these biomarker-driven strategies are translated into practice.

Concurrent ESR1 and *PIK3CA* mutations. The co-occurrence of ESR1 and *PIK3CA* mutations in the same tumor is increasingly identified through comprehensive ctDNA profiling and presents a clinical challenge, as both mutations predict resistance to different endocrine backbones (AIs for ESR1, fulvestrant-based regimens for *PIK3CA*-directed strategies). No randomized data exist specifically addressing this co-mutated population. For patients meeting INAVO120 criteria, the inavolisib triplet may be preferred regardless of ESR1 co-mutation, as inavolisib’s PI3Kα inhibition acts downstream of both alterations, and the fulvestrant backbone addresses ESR1-associated AI resistance. For patients receiving ctDNA-guided surveillance on AI plus CDK4/6 inhibitor, emergence of an ESR1 mutation may prompt a switch to a next-generation oral SERD plus CDK4/6 inhibitor (per SERENA-6), with consideration of PI3Kα-directed intensification at subsequent progression. Prospective trials specifically addressing co-mutated populations are urgently needed [84].

CDK4/6 inhibitor choice in the context of inavolisib. Ribociclib is currently favored by many clinicians and guidelines as the preferred CDK4/6 inhibitor based on consistent OS data across the MONALEESA program. However, INAVO120 used palbociclib as the backbone. Whether inavolisib combined with ribociclib or abemaciclib would yield equivalent or superior results is unknown, and no cross-trial comparison is valid given differences in patient populations. Clinicians must currently choose between adhering to the trial-validated palbociclib-based triplet or substituting ribociclib (off-label, without direct evidence). In the absence of head-to-head data, clinical judgment, institutional experience, and individual patient factors, including comorbidities, prior treatment exposure, and tolerability concerns, should guide this decision.

Readiness of serial ctDNA testing for community practice. Despite the transformative findings of PADA-1 and SERENA-6, implementation of serial ctDNA-guided surveillance in community oncology settings faces significant barriers. These include the following: (1) cost and reimbursement uncertainty for serial liquid biopsy testing; (2) absence of universally validated and standardized assays for ESR1 mutation quantification; (3) testing frequency optimization, both trials tested approximately every 2–3 months aligned with imaging schedules, but adherence to such protocols may be lower in routine practice; (4) provider and patient education requirements. While ctDNA-guided therapy represents the future standard, broader adoption will require regulatory endorsement of validated commercial assays, payer coverage decisions, prospective real-world implementation studies, and clarification of cost-effectiveness.

### 5.4. Biomarker-Guided Preemptive Therapy

Advances in genomic profiling (e.g., *PIK3CA* mutations, *ESR1* mutations, PTEN loss) increasingly inform individualized therapy choices. Liquid biopsy using circulating tumor DNA (ctDNA) enables real-time assessment of mutational status, guiding therapy selection and monitoring resistance [95].

A growing body of evidence supports the clinical utility of ctDNA-guided therapy to detect emerging and adapt endocrine treatment in HR–positive, HER2-negative MBC [96,97].

The PADA-1 trial provided the first prospective, randomized evidence that ctDNA-guided endocrine switching can improve treatment outcomes in such patients. In this phase 3 study, 1017 patients receiving first-line AI plus palbociclib underwent serial plasma monitoring for emergent ESR1 mutations. Among the 279 (27.4%) patients who developed a rising ESR1 mutation in ctDNA in the absence of radiologic progression, 172 (16.9%) were randomized to either switch early to fulvestrant plus palbociclib or to continue AI plus palbociclib until disease progression. The trial demonstrated that preemptive endocrine switching significantly prolonged PFS from the time of randomization, with a median PFS of 11.9 months in the fulvestrant–palbociclib group versus 5.7 months in those who continued AI–palbociclib (HR 0.61; 95% CI 0.43–0.86; *p* = 0.005). Importantly, this benefit was achieved without waiting for clinical or radiologic progression, highlighting that molecular progression detected by ctDNA precedes overt disease progression and is clinically actionable. PADA-1 therefore established a new paradigm in which liquid biopsy-based surveillance enables early identification of endocrine resistance and timely adaptation of therapy, effectively doubling disease control compared with standard radiology-guided management and providing a foundation for next-generation ctDNA-driven strategies [98,99].

Building on the findings of PADA-1, the SERENA-6 Phase 3 trial investigated a prospective ctDNA surveillance strategy to detect emergent ESR1 mutations before radiologic progression and to guide early therapeutic switching in first-line HR-positive, HER2-negative advanced breast cancer [84]. In this study, over 3250 patients receiving an AI plus a CDK4/6 inhibitor were monitored with ctDNA testing every 2–3 months coinciding with routine imaging until 315 patients with newly detected ESR1 mutations, but without evidence of radiologic progression, were randomized 1:1 to either switch to the next-generation oral SERD camizestrant (75 mg daily) plus continued CDK4/6 inhibition (n = 157) or to continue AI plus the same CDK4/6 inhibitor (n = 158). At the prespecified interim analysis, camizestrant demonstrated a highly statistically significant and clinically meaningful improvement in PFS, with a median PFS of 16.0 months (95% CI, 12.7–18.2) versus 9.2 months (95% CI, 7.2–9.5) for the AI arm, corresponding to a 56% reduction in the risk of progression or death (HR 0.44; 95% CI, 0.31–0.60; *p* < 0.00001). The benefit was consistent across all analyzed subgroups irrespective of the type of CDK4/6 inhibitor used. Landmark PFS rates further illustrated this effect: at 12 months, the PFS rate was 60.7% with camizestrant versus 33.4% with continued AI, and at 24 months it remained 29.7% versus 5.4%, respectively. Exploratory quality-of-life assessments showed that switch to camizestrant was associated with a marked delay in deterioration of global health status, with a median time to quality-of-life decline of 23.0 months compared with 6.4 months for the AI arm (HR 0.53; 95% CI, 0.33–0.82). Overall survival and additional secondary analyses remain immature at the current cut-off. Across both arms, treatment was generally well tolerated with low discontinuation rates for adverse events, and safety profiles consistent with known effects of endocrine therapy and CDK4/6 inhibition [83]. These data establish SERENA-6 as the first pivotal demonstration that ctDNA-guided early intervention with a next-generation SERD markedly improves disease control in patients with emergent endocrine resistance, reinforcing the clinical value of real-time molecular monitoring to optimize first-line management in HR+ metastatic breast cancer [92]. Table 4 summarizes clinical trials that utilize the ctDNA preemptive approach.

## 6. Conclusions and Future Directions

The therapeutic landscape of HR-positive, HER2-negative MBC has undergone a profound transformation, with CDK4/6 inhibitors now firmly established as the backbone of first-line therapy in combination with endocrine treatment [33,34]. Agents such as ribociclib have demonstrated consistent and clinically meaningful overall survival benefits across multiple disease settings, including patients with visceral metastases and aggressive clinical features, redefining standards of care and enabling chemotherapy-sparing strategies for most patients [37,48,58,60].

Looking forward, the field is rapidly moving beyond sequential, fixed treatment algorithms toward an adaptive model and precision-guided care [100]. The emergence of next-generation ER–targeted agents, particularly SERDs, offers the opportunity to more effectively suppress ER signaling and overcome ESR1 mutation-driven resistance [101]. Additionally, targeted pathway inhibition, most notably PI3Kα inhibitors in *PIK3CA*-mutated disease, enables more durable blockade of key oncogenic drivers when combined with ET and CDK4/6 inhibitors.

**Figure 1:**
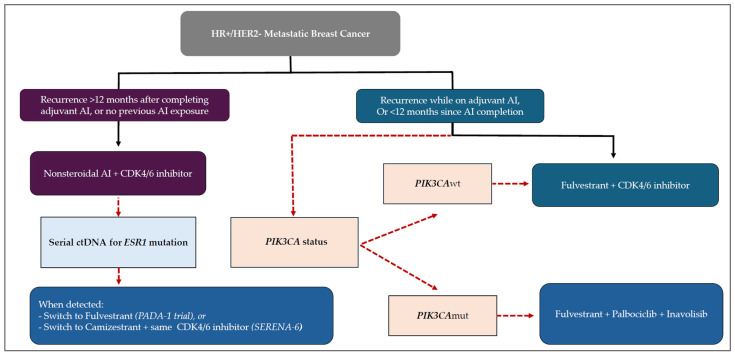
Suggested algorithm for management of patients with HR+/HER2− MBC treated in the first-line setting. Black solid lines indicate the accepted standard of care; dashed red lines indicate potential new standards. MBC: Metastatic Breast Cancer; HR+: Hormone Receptor-positive; HER2−: Human Epidermal Growth Factor-2 Negative; AI: Aromatase inhibitors; ctDNA: Circulating DNA; *PIK3CA*mut: *PIK3CA*-mutated; *PIK3CA*wt: *PIK3CA* wild-type.

However, the most transformative development in this field is the integration of ctDNA-based liquid biopsy into routine breast cancer clinical practice [11]. Real-time molecular surveillance allows early detection of emerging resistance mechanisms and enables proactive therapeutic adaptation, as described by the PADA-1 and SERENA-6. These studies provide proof of concept that endocrine switching based on ctDNA detection can extend disease control beyond the classical imaging-based strategies, Figure 1 [102,103].

While this review focuses on first-line therapy, it is important to acknowledge that the treatment continuum for HR+/HER2− MBC is increasingly shaped by advances in later-line settings. Antibody–drug conjugates (ADCs), in particular trastuzumab deruxtecan for HER2-low and HER2-ultralow disease, and sacituzumab govitecan, have emerged as active agents in heavily pre-treated HR+ populations and are influencing long-term sequencing decisions [104,105]. These agents are not first-line therapies, but their potential use in later lines reinforces the importance of comprehensive molecular profiling at baseline, including HER2 expression by IHC. In the realm of targeted therapy, AKT pathway inhibition with capivasertib plus fulvestrant (CAPItello-291) represents an important pathway-directed strategy for patients with HR+/HER2− MBC who have progressed after prior endocrine therapy. Finally, beyond first-line treatment, emerging data from trials such as postMONARCH, MAINTAIN, and PACE are informing strategies for CDK4/6 inhibitor re-challenge and sequencing after progression, and readers are referred to dedicated reviews of this evolving landscape [106,107,108].

## Figures and Tables

**Table 1:** Summary of clinical trials using CDK4/6 inhibitors in the first-line setting for HR+/HER2- advanced-stage breast cancer.

	PALOMA-2 [48] (N = 666)	MONARCH-3 [49,50] (N = 493)	MONALEESA-2 [51] (N = 668)	MONALEESA-3 [53] (N = 726) †	MONALEESA-7 [57,58] (N = 672)
Line of therapy	First-line	First-line	First-line	First and second line	First-line
Patient population	Postmenopausal advanced BC; no previous systemic treatment	Locally advanced or metastatic BC; postmenopausal; no previous systemic treatment	Postmenopausal advanced BC with recurrent or MBC; no previous systemic treatment	Postmenopausal advanced BC; ≤1 prior ET for advanced BC	Premenopausal/ perimenopausal advanced BC; no previous ET for advanced BC
Treatment arms	Letrozole ± palbociclib	Anastrozole or letrozole ± abemaciclib	Letrozole ± ribociclib	Fulvestrant ± ribociclib	Tamoxifen, anastrozole, or letrozole ± ribociclib
Median PFS CDK4/6i + ET vs. ET, months	27.6 vs. 14.5 (HR: 0.56)	29.0 vs. 14.8 (HR: 0.54)	25.3 vs. 16.0 (HR: 0.57)	20.5 vs. 12.8 (HR: 0.59)	23.8 vs. 13.0 (HR: 0.55)
Median OS CDK4/6i + ET vs. ET, months	53.9 vs. 51.2 (HR: 0.96)	66.8 vs. 53.7 (HR: 0.80)	63.9 vs. 51.4 (HR: 0.76)	67.6 vs. 51.8 (HR: 0.67)	58.7 vs. 48.0 (HR: 0.76)

† MONALEESA-3 enrolled patients who had received up to one prior line of ET for advanced disease (both first-line and second-line patients), in contrast to all other trials in this table, which enrolled exclusively first-line patients. This enrollment heterogeneity should be considered when interpreting the OS and PFS results from MONALEESA-3 in a pure first-line context. BC: breast cancer; CDK4/6i: cyclin-dependent kinase 4/6 inhibitor; ET: endocrine therapy; HER2-: Human epidermal growth factor receptor 2–negative; HR: Hazard ratio; HR+: Hormone receptor-positive; MBC: Metastatic breast cancer; OS: overall survival; PFS: progression-free survival.

**Table 2:** Summary of clinical trials utilizing CDK4/6 inhibitors in patients with HR+/HER2− advanced-stage breast cancer with visceral metastasis.

Feature	RIGHT Choice [60]	PADMA [61] *	ABIGAIL [63,64]
Phase/Design	Phase 2, randomized	Phase 3, randomized	Phase 2, randomized
Patient population	Pre/perimenopausal women with clinically aggressive MBC, including symptomatic visceral metastases, visceral crisis, or rapid progression	MBC with indication for chemotherapy (e.g., visceral metastases, rapid progression)	MBC with ≥1 aggressive feature: symptomatic visceral disease, liver metastases, ≥3 metastatic sites, or early relapse
CDK4/6 inhibitor	Ribociclib	Palbociclib	Abemaciclib
Comparator	Physician’s choice combination chemotherapy	Mono-chemotherapy	Paclitaxel (12 weeks induction)
Endocrine therapy backbone	Letrozole or anastrozole + goserelin	ET (varied)	Standard ET
Number of patients	222	130 (120 included in the analysis)	162
Primary endpoint	PFS	TTF/PFS	12-week objective response rate (ORR)
Median PFS (Months)	21.8 (Ribociclib + ET) vs. 12.8 (CT), HR 0.61, *p* = 0.003	18.7 vs. 7.8 HR 0.45, 95% CI 0.29–0.70, *p* < 0.001	Not powered for PFS (ORR-driven)
ORR (%)	66.1 (Ribociclib + ET) vs. 61.8 (CT)	Not reported	58.8 (Abemaciclib + ET) vs. 40.2 (Paclitaxel) [OR 2.11; 95% CI, 1.13–3.96; *p* = 0.0193]
Tolerability	Fewer symptomatic AEs and fewer discontinuations with Ribociclib + ET	Better tolerability than chemotherapy	Similar safety, trend toward better tolerability with Abemaciclib + ET

AEs: Adverse events; MBC: Metastatic breast cancer; CDK4/6i: cyclin-dependent kinase 4/6 inhibitor; ET: Endocrine therapy; HER2−: Human epidermal growth factor receptor 2–negative; HR: Hazard ratio; HR+: Hormone receptor-positive; MBC: Metastatic breast cancer; NS: Not significant; OR: Odds ratio; ORR: Objective Response Rate; OS: overall survival; TTF: Time to Treatment Failure; PFS: progression-free survival. * Full publication is pending.

**Table 3:** Clinical outcomes of patients treated with inavolisib in addition to palbociclib and fulvestrant.

Outcome	Inavolisib + Palbociclib + Fulvestrant	Placebo + Palbociclib + Fulvestrant	Treatment Effect
Number of patients	161	164	—
Median follow-up (Months)	34.2	32.3	—
Median PFS (Months)	17.2	7.3	HR 0.42 (95% CI: 0.32–0.55)
Median Overall Survival (OS) (Months)	34.0 (95% CI, 28.4–44.8)	27.0 (95% CI, 22.8–38.7)	HR 0.67 (95% CI: 0.48–0.94), *p* = 0.02
Objective Response Rate (ORR)	62.7% (95% CI, 54.8–70.2)	28.0% (95% CI, 21.3–35.6)	*p* < 0.0001
Median Time to Chemotherapy (Months)	35.6 (95% CI, 25.4–NR)	12.6 (95% CI, 10.4–16.1)	HR 0.43
Median duration of response (Months)	19.2 (95% CI, 14.7–28.3)	11.1 (95% CI, 8.5–20.2)	—
Discontinuation due to AEs	6.8%	0.6%	—

NR: Not reached; CI: Confidence Interval; HR: Hazard Ratio.

**Table 4:** Summary of clinical trials that utilized a preemptive approach with serial ctDNA testing for *ESR1* mutation.

Feature		PADA-1 [98,99]	SERENA-6 [84]
Design		Phase 3, open-label, randomized	Phase 3, double-blind, randomized
Population		HR+/HER2− MBC on AI + palbociclib with emergent *ESR1* mutation in ctDNA but no radiologic progression	HR+/HER2− MBC on AI + CDK4/6 inhibitor with emergent *ESR1* mutation in ctDNA (prospective monitoring) and no radiologic progression
Randomization trigger		Rising *ESR1* mutation detected before imaging progression	Newly detected *ESR1* mutation during routine ctDNA surveillance
Study Arms	Experimental	Switch to fulvestrant + palbociclib	Switch to camizestrant + same CDK4/6 inhibitor
	Control	Continue AI + palbociclib	Continue AI + same CDK4/6 inhibitor
Primary endpoint		PFS from randomization at *ESR1* detection	PFS
Median PFS		11.9 months (switch) vs. 5.7 months (continue AI)	16.0 months (camizestrant switch) vs. 9.2 months (continue AI)
Hazard ratio (PFS)		0.61 (significant benefit for early switch)	0.44 (highly significant benefit for early switch)
Key clinical message		Early ctDNA-guided switch to fulvestrant upon *ESR1* emergence doubles PFS vs. waiting for radiologic progression	Prospective ctDNA surveillance enabling early switch to a next-gen SERD (camizestrant) provides substantial PFS gain and delays clinical progression

AI: Aromatase inhibitors; ctDNA: Circulating DNA; HER2−: Human epidermal growth factor receptor 2–negative; HR: Hazard ratio; HR+: Hormone receptor-positive; MBC: Metastatic breast cancer; PFS: Progression-free survival.

## Data Availability

No new data were created or analyzed in this study. Data sharing is not applicable to this article.
